# Assessment of ductal blood flow in newborns with obstructive left heart lesions by cardiovascular magnetic resonance

**DOI:** 10.1186/1532-429X-15-45

**Published:** 2013-05-28

**Authors:** Marcelo Felipe Kozak, Shi-Joon Yoo, Luc Mertens, Ashley Ho, Lars Grosse-Wortmann

**Affiliations:** 1 Labatt Family Heart Centre in the Department of Paediatrics and Department of Diagnostic Imaging, The Hospital for Sick Children, University of Toronto, Toronto, ON, Canada

**Keywords:** Patent ductus arteriosus, Flow, Cardiovascular magnetic resonance, Obstructive, Newborn, Congenital heart disease

## Abstract

**Background:**

Newborns with obstructive left heart lesions often depend on a patent ductus arteriosus to sustain the systemic circulation. Our aims were to validate the direct measurement of ductal flow, and to characterize the magnitude, determinants and hemodynamic effects of patent ductus arteriosus in newborns with obstructive left heart lesions by cardiovascular magnetic resonance (CMR).

**Methods:**

In this retrospective study, the CMR and clinical information of newborns with obstructive left heart lesions were reviewed. The feasibility and validity of measuring ductal flow and the correlations between ductal flow and ventricular volumes, ascending aortic flow, post-ductal oxygen saturation and Qp:Qs were assessed.

**Results:**

The CMR examinations of 32 newborns were included. It was possible to measure the ductal flow in all of them, with moderate-to-good agreement between measured and calculated ductal flow volume. The flow was bidirectional in all patients, with a net right-to-left shunt in 72%. Net ductal flow correlated inversely with ascending aortic flow (Rho −0.63; p 0.0002), post-ductal oxygen saturation (Rho −0.58; p 0.0004), Qp:Qs (Rho −0.43; p 0.02), and with left ventricular end-diastolic volume index (Rho −0.38; p 0.04). There was no correlation with the diameter of the ductus. The contribution of ductus flow to the systemic circulation correlated with the left ventricular end-diastolic volume index (Rho −0.47; p 0.02).

**Conclusions:**

Direct measurement of ductal flow in newborns with obstructive left heart lesions is feasible. From these measurements, we were able to demonstrate that patients with smaller left ventricles and lower ascending aortic flow have a greater contribution of ductal flow to the systemic circulation. The size of the ductus arteriosus does not predict net ductal flow. Phase-contrast CMR can be an adjunct method for the assessment of the physiology for very ill neonate patients.

## Background

Newborns with significant congenital obstructive left heart lesions (OLHL), including hypoplasia of the left ventricle (LV), severe aortic valve stenosis and aortic arch obstructions, frequently rely on the contribution of blood flow via the patent ductus arteriosus (PDA) to the systemic circulation. In the medical and surgical management of these patients it is important to know to what extent the systemic circulation depends on the contribution from flow through the PDA and what the relation of pulmonary to systemic blood flow (Qp:Qs) is [1-3].

In clinical practice, shunt magnitude and net flow direction are typically estimated by semiquantitative ultrasound assessment, including measurements of ductal size, “eyeballing” of the color Doppler profile, and interpretation of pulsed or continuous wave Doppler flow profiles [4]. Velocity mapping using “through-plane” phase contrast cardiovascular magnetic resonance (PC-CMR) is the gold-standard for non-invasive flow assessment [5-7]. Any adequately sized vessel in any orientation, including the arterial duct, can be evaluated for blood flow velocity, volume and pattern. PC-CMR has been routinely used in patients with congenital heart diseases for the assessment of blood flow, including shunting quantification [5,8].

The aims of the current study were to validate the direct measurement of ductal flow, characterize the magnitude, the determinants and the hemodynamic and clinical effects of flow through the PDA in newborns with OLHL by CMR.

## Methods

Following approval by the Hospital for Sick Children research ethics board, the imaging studies and clinical information of all newborns with OLHL who were on a prostaglandin infusion and underwent CMR during the first month of life, prior to any kind of intervention to alleviate the obstruction between August 2003 and July 2011 were retrospectively reviewed.

CMR was performed on a 1.5 Tesla scanner (“GE signa CV/*I*”, General Electric Medical Systems, Milwaukee, WI, USA, before July 2007; and “Avanto”, Siemens Medical Solutions, Erlangen, Germany, thereafter), using head-and-neck coils. PC-CMR datasets were acquired using retrospective ECG-gating, during free-breathing or continued mechanical ventilation under sedation, and applying the following settings: Slice thickness (4 mm), in-plane spatial resolution of 1 × 1 mm, temporal resolution sufficient to acquire at least 20 true phases per cardiac cycle, 2 averages, minimum echo and repetition times, and flip angle of 15–30°. The velocity encoding limit varied between 150–400 cm/s and was chosen according to the vessel of interest: lower velocities for veins and higher velocities for arteries. The typical acquisition time for each PC-CMR sequence scan time was 90 seconds.

**Figure 1:**
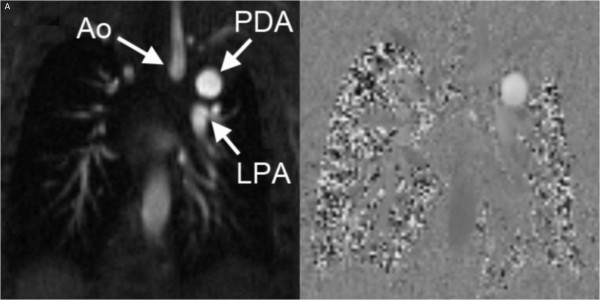
Magnitude and phase images of through-plane phase-contrast imaging of the patent ductus arteriosus (PDA) (Ao = transverse aortic arch; LPA = left pulmonary artery).

**Figure 2:**
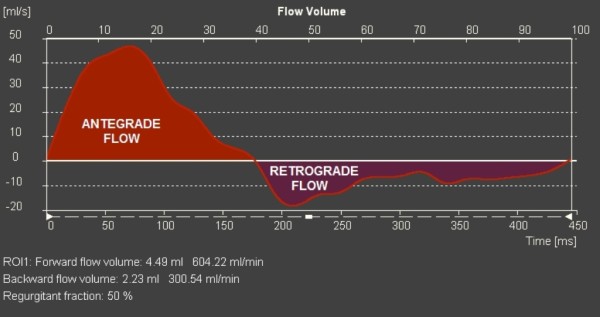
Flow volume curve across the patent ductus arteriosus in a patient with mitral stenosis and aortic valve atresia, showing bidirectional flow.

PDA flow was captured by acquiring PC-CMR velocity data in the short axis of the vessel (Figures 1 and 2). The imaging slice was positioned using multiplanar reformats of a three-dimensional contrast-enhanced angiographic dataset. In addition, flows in the superior vena cava (SVC); ascending (AAo) and descending aorta (DAo); main pulmonary artery (MPA), left (LPA) and right (RPA) branch pulmonary arteries were obtained. For the purpose of this study, all flows were reanalyzed on a commercially available work station (QFlow 5.2; Medis Medical Imaging Systems, Leiden, The Netherlands). The durations of antegrade (right-to-left) and retrograde (left-to-right) flows across the PDA were measured from the flow-time curves generated from the PC-CMR data. Their relation was termed antegrade/retrograde duration ratio (AR ratio).

These measurements were used for calculating systemic (Qs) and pulmonary (Qp) flows, using the following equations:

(1) Qs=SVC+DAo

(2) Qp=RPA+LPA

In order to internally validate the measured PDA flow, it was also calculated by the following two methods:

(3) PDA=Qs+−AAo

(4) $$\mathrm{PDA}=\mathrm{MPA}–\left(\mathrm{Qp}\right)$$

The largest ductal diameter during the cardiac cycle was measured on cross-sectional cine images. Left and right ventricular volumes and ejection fractions were measured from a stack of short axis cine images which were analyzed using commercially available software (Mass Analysis and QMass 7.2, both Medis Medical Imaging Systems). Flow and volumetric measurements were standardized to body surface area using Mosteller’s formula: BSA = {[Height (cm) × Weight (Kg)]/3600}^1/2^. By definition, negative flow denoted a left-to-right flow across the PDA.

Patients’ demographics and clinical information were retrieved from the medical records.

### Statistical analyses

All statistical analyses were conducted using the software StatsDirect, version 2.7.2. 2008 (Cheshire, United Kingdom).

Spearman’s rank correlation (Rho) was calculated to assess the relation between flow measurements by PC-CMR and clinical variables. *T-tests* were used to compare continuous numeric data of subgroups. Categorical variables were expressed using frequency distribution and percentages, and comparisons were made using the Fisher exact test. Bland-Altman analyses were used to express the agreement between direct measurements of PDA flow and calculated PDA flow as per Equations 3 and 4. P values < 0.05 were considered significant.

## Results

Thirty-two newborns with OLHLs were included. The patients’ demographic information and clinical parameters are outlined in Table 1. All patients received a contiuous infusion of prostaglanding during the examination. In 25 out of the 32 patients the exam was performed under mechanical ventilation and general anesthesia. Five were sedated with propofol, but not intubated and two underwent a feed-and-sleep approach. There were 23 cases with mitral valve stenosis and / or mitral hypoplasia. Of these 23, 19 had aortic valve stenosis, 16 had coarctation of the aorta, four had aortic valve atresia, and four had ventricular septal defects. One patient had mitral and aortic valve atresia and one patient had an unbalanced atrioventricular septal defect with right ventricular dominance and hypoplastic aortic arch. Of the remaining seven patients with normal mitral valves, five had aortic valve stenosis (three with coarctation of the aorta and one with type B interruption of the aortic arch), one case had isolated coarctation of the aorta and one presented with an obstructive tumor in the LV. The reasons for CMR were: to assess the volume of the LV and antegrade flow through the AAo for surgical decision-making between bi- and univentricular repair in 28 cases, to assess the morphology and extent of a cardiac tumor in one case, to aid in planning balloon dilatation of the aortic valve in one case, to better define the pulmonary venous connection in one case and to better visualize the aortic arch in another case.

**Table 1:** Demographic and clinical information

	**All patients (n = 32)**
Gender	21 male (65.6%)
Age	4.3 ± 3.2 (0–13) days
Weight	3.12 ± 0.57 (1.70 – 4.44) Kg
Body surface area	0.21 ± 0.03 (0.14 – 0.26) m^2^
PDA diameter	7.0 ± 1.6 (3.8 – 11.5) mm
Post-ductal O_2_ saturation	91 ± 6 (74 – 99)%
Heart rate	131 ± 17 (98 – 164) bpm

*PDA* patent ductus arteriosus.

**Figure 3:**
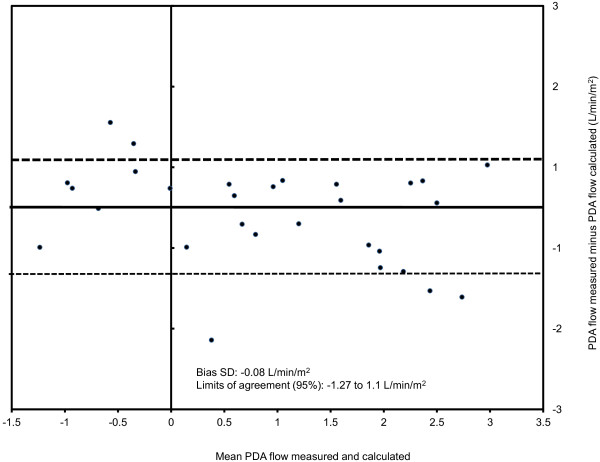
**Bland-Altmann plot for PDA flow measured and calculated as per Equation** 3 **(PDA = Qs – AAo).**

**Figure 4:**
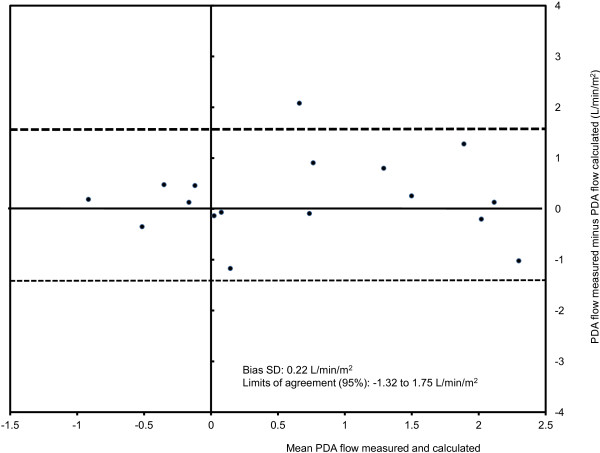
**Bland-Altmann plot for PDA flow measured and calculated as per Equation** 4 **(PDA = MPA – QP).**

CMR measurements are presented in Table 2. The flow was bidirectional in all patients, and net PDA flow was from right-to-left in the majority of patients (23/32, 72%). It was possible to quantify the PDA flow in all patients. Measured PDA flow correlated very well with the calculated PDA flow using Equation 3 (Rho = 0.90, p < 0.0001), and moderately well with the calculated PDA flow using Equation 4 (Rho = 0.73, p = 0.001). However, by using Bland-Altman analysis the variability of measurements were found wide by both Equation 3 (Bias SD = −0.08 L/min/m^2^; limits of agreement 95% = −1.27 to 1.10 L/min/m^2^, Figure 3) and Equation 4 (Bias SD = 0.22 L/min/m^2^; limits of agreement 95% = −1.32 to 1.75 L/min/m^2^, Figure 4).

**Table 2:** CMR findings

**Variables**	**All patients**	**Patients with net RL shunt**	**Patients with net LR shunt**	**P**
LVEDVi (ml/m^2^)	25.8 ± 10.9 (11.8 - 71.2)	24.9 ± 12.6 (11.8 - 71.2)	27.8 ± 5.5 (21–35.2)	0.40
RVEDVi (ml/m^2^)	77.3 ± 26.4 (41.6 – 140.5)	81.8 ± 26.8 (47 – 140.5)	66.3 ± 23.6 (41.6 – 106)	0.16
LV/RV volume	0.39 ± 0.27 (0.1 - 1.51)	0.36 ± 0.3 (0.1 - 1.51)	0.47 ± 0.16 (0.26 – 0.71)	0.33
RVEF (%)	49.1 ± 9.1% (28 – 67)	49 ± 8.8% (28 – 62)	49.3 ± 10.7% (31–53)	0.94
LVEF (%)	59.4 ± 11.5% (36 – 81)	58.2 ± 11.1% (36 – 81)	62.3 ± 12.7% (36 – 77)	0.38
PDA flow* (L/min/m^2^)	0.86 ± 1.20 (−1.48 – 3.24)	1.44 ± 0.83 (0.04 – 3.24)	−0.62 ± 0.49 (−1,48 – -0.04)	< 0.0001
AAo flow (L/min/m^2^)	1.39 ± 0.99 (−0.19 – 2.96)	1.08 ± 0.85 (−0.19 – 2.41)	2.24 ± 0.88 (0.32 – 2.96)	0.003
SVC flow (L/min/m^2^)	1.37 ± 0.5 (0.46 – 2.93)	1.5 ± 0.51 (0.63 – 2.93)	1 ± 0.23 (0.46 – 1.22)	0.001
DAo flow (L/min/m^2^)	1.03 ± 0.48 (0.3 – 2.42)	1.13 ± 0.5 (0.3 – 2.42)	0.76 ± 0.26 (0.41 – 1.24)	0.06
Pulmonary flow (RPA + LPA) (L/min/m^2^)	4.22 ± 1.79 (1.62 – 7.39)	4.14 ± 1.71 (1.62 – 7.14)	4.41 ± 2.1 (1.82 – 7.39)	0.72
Systemic flow (SVC + DAo) (L/min/m^2^)	2.4 ± 0.94 (0.93 – 5.35)	2.66 ± 1 (0.93 – 5.35)	1.75 ± 0.23 (1.41 – 2.05)	0.02
Qp:Qs	2.2 ± 1.34 (0.38 – 5.82)	2.04 ± 1.36 (0.38 – 5.82)	2.57 ± 1.29 (0.89 – 4.26)	0.36
Contribution of PDA to Qs (%)	27.7 ± 48.3% (−87^†^ – 100)	52.5 ± 26.8% (4.7 – 100)	−34.2 ± 29.9% (−87.2 – -2.39)	< 0.0001
Contribution of PDA to DAo (%)	41.7 ± 74.7% (−100^†^ – 100)	81.7 ± 31.9% (5.2 – 100)	−63.2 ± 44.9% (−100 – -5.29)	< 0.0001
Upper body flow%	57.7 ± 8.2% (27.1 – 70.9)	57 ± 5.3% (46.7 – 67.7)	60.5 ± 13.4% (27 – 70.9)	0.96
AR ratio	0.59 ± 0.17 (0.28 – 0.92)	0.66 ± 0.14 ( (0.45-0.92)	0.40 ± 0.09 (0.28 – 0.52)	< 0.0001

*negative values denote a left-to-right shunt.

^†^negative percentages denote a contribution of aortic arch flow to pulmonary blood flow via a left-to-right shunt across the PDA.

*AAo* ascending Aorta, *AR ratio* antegrade/retrograde duration ratio, *DAo* descending Aorta, *LPA* left pulmonary artery, *LV* left ventricle, *LVEDVi* LV end-diastolic volume index, *LVEF* LV ejection fraction, *PDA* patent ductus arteriosus; *Qp:Qs* pulmonary flow: systemic flow, *RPA* right pulmonary artery, *RV* right ventricle, *RVEDVi* RV end-diastolic volume index, *RVEF* RV ejection fraction, *SVC* superior vena cava.

PDA net flow correlated strongly with the AR ratio (Rho = 0.88; p < 0.0001), inversely with the amount of AAo flow (Rho = −0.63; p = 0.0002), post-ductal oxygen saturation (Rho = −0.58; p = 0.0004), Qp:Qs (Rho = −0.43; p = 0.02), and LVEDVi (Rho = −0.38; p = 0.04). There were no statistically significant correlations of PDA net flow volume with PDA diameter, whether the latter was indexed or not to body surface area (Rho = 0.26; p = 0.15 for both) or right ventricular end-diastolic volume index (Rho = 0.21; p = 0.29).

Three patients did not undergo ventricular volumetry. Out of the other 29 patients, seven had a LVEDVi of less than 20 ml/m^2^. All patients with LVEDVi < 20 ml/m^2^ presented with a net right-to-left flow across the PDA, while nine out of the other 22 patients (41%) had a net left-to-right flow across the PDA (p = 0.05). Patients with LVEDVi < 20 ml/m^2^ had a greater mean right-to-left shunt than patients with larger LV’s (1.65 ± 0.49 L/min/m^2^ vs 0.46 ± 1.21 L/min/m^2^, respectively, p = 0.001) as well as a greater contribution of the PDA flow to the systemic circulation than those with bigger ventricles (61.7 ± 19.3%% vs 16.4 ± 50.0%; p = 0.002). LVEDVi correlated with ascending aortic flow (Rho = 0.44; p = 0.02).

The contribution of PDA flow to the systemic circulation varied between −87% and 100% (Table 2; negative values represent left-to-right shunting across the PDA). There was a strong positive correlation between this contribution and the AR ratio (Rho = 0.83; p < 0.0001), and a negative correlation between this contribution and flow in the ascending aorta (Rho = −0.73; p < 0.0001), and also with LVEDVi (Rho = −0.47; p = 0.02). There was no correlation between the contribution of the PDA flow to the systemic circulation and PDA diameter, either in absolute diameter measurements (Rho = 0.21; p = 0.26) or indexed to body surface area (Rho = 0.17; p = 0.39).

Post-ductal oxygen saturation correlated with Qp:Qs (Rho = 0.49; p = 0.009) and inversely with the contribution of PDA flow to the descending aortic flow (Rho = −0.45; p = 0.008). Relative upper body perfusion, measured as the percentage of superior vena cava flow over total systemic flow, did not correlate with PDA net flow (Rho = −0.26; p = 0.17), or with antegrade ascending aortic flow (Rho = −0.09; p = 0.64).

An AR ratio ≥ 0.45 identified cases with a net right-to-left shunt with a sensitivity of 100% and a specificity of 88%. For an AR ratio ≥ 0.53 the sensitivity and specificity were respectively 79.2% and 100%.

In the subgroup of 23 patients with a net right-to-left shunt, ductal flow correlated weakly with ascending aortic flow (Rho = −0.43; p = 0.04), and PDA diameter (Rho = 0.42; p = 0.04). There was no correlation between the contribution of the PDA flow to the systemic circulation and PDA diameter (Rho = 0.26; p = 0.31).

Following CMR, 6 patients underwent a staged univentricular repair while a biventricular circulation was targeted in 24. Two received compassionate care. CMR results for the univentricular and biventricular repair groups, respectively are presented in Table 3. Patients who went on a univentricular repair relied more on contributions to their systemic circulation via right-to-left shunting across the PDA and had smaller left ventricles than those who underwent a biventricular repair. At the time of manuscript completion, 25 of 32 were alive. Of the 7 patients who died, 2 had received compassionate care, 3 had been treated with a univentricular and 2 with a biventricular approach.

**Table 3:** CMR findings of patients according to the type of treatment: univentricular or biventricular

**Variables**	**Univentricular**	**Biventricular**	**P**
PDA flow* (L/min/m^2^)	1.70 (0.56 to 2.53)	0.61 (−1.48 to 3.23)	0.0147
AAo flow (L/min/m^2^)	0.56 (−0.19 to 1.08)	1.82 (−0.16 to 2.92)	0.0006
Contribution of PDA to Qs (%)	56.59 (39.77 to 100)	24.14 (−87.16 to 100)	0.0013
LVEDVi (ml/m^2^)	15.75 (11.8 to 30.3)	27 (17.7 to 71.2)	0.024
AR ratio	0.72 ± 0.14 (0.52 to 0.92)	0.54 ± 0.17 (0.28 to 0.89)	0.01

*negative values denote a left-to-right shunt.

^†^negative percentages denote a contribution of aortic arch flow to pulmonary blood flow via a left-to-right shunt across the PDA.

*AAo* ascending Aorta, *AR ratio* antegrade/retrograde duration ratio, *LVEDVi* LV end-diastolic volume index, *PDA* patent ductus arteriosus.

## Discussion

Many patients with OLHL depend on PDA flow to sustain, partially or completely, the systemic circulation. Consequently, the ability to assess not only the cardiac anatomy, but also the functional status of the ductus arteriosus is important in the care of these patients. With this study we introduce direct measurements of ductal flow by PC-CMR and examine the determinants of ductal blood flow amount.

### PDA flow can be measured by CMR

In a study limited to systemic perfusion in preterm and term newborns, Groves and colleagues obtained more complete data with a greater reproducibility using CMR than existing echocardiographic methods [9]. In simple intracardiac shunt lesions, PC-CMR based quantification of Qp:Qs has shown excellent agreement with invasive oximetry [10-12]. Selective vessel assessments allow for shunt quantification in complex cases of congenital heart diseases, including patients with univentricular physiology [13-16], thus providing key information for invasive management decisions in very ill neonates [17].

In principle, it is possible to derive PDA flow in three different ways: 1) By direct measurement, 2) as the difference between LV cardiac output (AAo flow) and perfusion to the upper (SVC flow) and lower body (DAo flow), and 3) by subtracting branch pulmonary artery flows from main pulmonary artery flow. Recently, Broadhouse and colleagues [18], studying otherwise healthy neonates, quantified PDA flow volume by subtracting ascending aortic flow from Qs, similar to Equation 3 used here. The authors judged direct measurement of PDA to be technically not feasible due to the variable and unfavorable morphology. However, in neonates with OLHL, the duct tends to be straight, of considerable length and often large. In our experience, prescription of the appropriate PC acquisition is typically straightforward, provided that thin enough slices are used. In the present study, the directly measured PDA flow correlated very well with the PDA flow derived using Equation 3 (PDA = SVC + DAo – AAo). The weaker correlation with Equation 4 [PDA = MPA – (RPA + LPA)], might be related to inaccuracy of the flow measurements in the large main pulmonary artery with high volume flow. Nevertheless, the wide limits of agreement reflect considerable differences in results using measured PDA flow on the one hand and calculated flow on the other. In the absence of an independent gold standard, it is difficult to be affirmative about the accuracy of either method. However, on PC-CMR acquisitions (Figure 1) the PDA appears similar to other arteries like the aorta and the branch pulmonary arteries. As such, assessment of PDA flow based on single, direct measurements may well be more accurate than a composite calculation from three different methods, each with its own errors.

### PDA flow direction and magnitude, and contribution of ductal flow to the systemic circulation

PDA flow was bidirectional in all patients, with a net right-to-left shunt in the majority. The contribution of the ductal flow to the systemic circulation was up to 100%. Interestingly, in nine patients the PDA net flow was from the systemic to the pulmonary circulation, which could have influenced the management by leading to cessation of the prostaglandin infusion, as the PDA was not needed to supplement the systemic circulation. The LVEDVi in these nine patients was significantly greater than in the other patients with a right-to-left shunt across the PDA (Table 2).

As expected, more right-to-left flow across the PDA resulted in lower post-ductal oxygen saturations and lower Qp:Qs. PDA flow also correlated inversely with antegrade flow through the ascending aorta, LV/RV ratio and LVEDVi. Specifically, patients with an LVEDVi of less than 20 ml/m^2^, one of the parameters commonly used in surgical decision making between uni- or biventricular repair [19,20], presented with a significantly greater amount of right-to-left flow through the PDA than those with bigger ventricles. Clinically and pathophysiologically, these findings feed into the expectation that patients with larger LVs and greater antegrade flow in the ascending aorta rely less on a contribution from PDA flow to the systemic circulation.

### PDA size is a poor predictor of shunt magnitude

Intuitively, a large caliber PDA suggests a large amount of flow. However, in the overall cohort of patients, we did not find a correlation between PDA diameter and shunt magnitude, or contribution of PDA flow to the systemic circulation. In the patients with a net right-to-left shunt across the duct its size correlated with the net shunt amount, but the correlation was weak and is unlikely to be helpful in gauging the PDA contribution to the systemic circulation in a clinical setting.

These results suggest that PDA size may be simply a consequence of pendular flow, i.e., alternating forward and backward flow, rather than net flow. The same mechanism of dilatation through pendular flow is found in patients with absent pulmonary valve syndrome.

There was a strong positive correlation of the duration of right-to-left (antegrade) flow, relative to the left-to-right flow duration, with PDA net right-to-left flow and with the contribution of PDA flow to the systemic flow. Hence, a longer relative antegrade flow duration allows more right-to-left shunt across the PDA. Even in the absence of a full hemodynamic assessment by CMR, the AR ratio may add to the identification of duct-dependency in patients with OLHL. We hypothesize that AR ratio, which is also obtainable by echocardiography, may become a suitable marker for LV output in patients with OLHL and for the degree of contribution of PDA flow to the systemic circulation.

### CMR results and patients outcomes

Although this was a retrospective study and not aimed at assessing the significance of PDA flow for patient outcomes, it is interesting to note that patients who went on to undergo a univentricular repair strategy depended more on PDA flow for their systemic perfusion, had a greater degree of right-to-left shunt and smaller LVs. As PDA flow was not directly used for surgical decision-making it is tempting to speculate that patients who have greater ascending aortic flow and rely less on PDA flow for Qs are more likely to be subject to a biventricular repair strategy.

### Study limitation

The limited cohort size may have obscured certain associations between parameters. As the data was collected retrospectively, the patient’s clinical data was limited to what was recorded at the time of CMR, as the hemodynamic physiology in OLHL is often labile and associations with clinical parameters outside of the CMR study were deemed to be unreliable. It would have been interesting to demonstrate that the AR ratio measured by CMR can be substituted by a similar measurement by Doppler echocardiography. However, our review of oxygen saturations and ventilation parameters showed that the physiology during the echocardiogram closest to the CMR was significantly different to that during the CMR study which prohibited a comparison of the two modalities.

## Conclusions

Direct and indirect assessments of ductal flow in newborns with obstructive left heart lesions are feasible. Patients with OLHL present with a wide spectrum of PDA flow magnitude. Those with smaller left ventricles and ascending aortic blood flow rely more on the contribution from PDA flow to the systemic circulation.

Ductal flow, and its contribution to the systemic circulation, cannot be inferred reliably from PDA size. In patients in whom net PDA flow is a component of medical or surgical decision making direct measurement of ductal blood flow by CMR should be considered.

## Abbreviations

AAo: Ascending aorta; AR ratio: Antegrade/retrograde duration ratio; CMR: Cardiovascular magnetic resonance; DAo: Descending aorta; LPA: Left pulmonary artery; LV: Left ventricle; LVEDVi: LV end-diastolic volume index; MPA: Main pulmonary artery; OLHL: Obstructive left heart lesions; PC-CMR: Phase contrast cardiovascular magnetic resonance; PDA: Patent ductus arteriosus; Qp: Pulmonary flow; Qs: Systemic flow; RPA: Right pulmonary artery; RV: Right ventricle; SVC: Superior vena cava.

## Competing interests

The authors declare that they have no competing interests.

## Authors’ contributions

MFK collected the data, performed the statistical analyses, and drafted the manuscript. SJY collected data and revised the manuscript. LM assisted in designing the study and revised the manuscript. AH collected clinical and CMR data. LGW designed the study, participated in data collection and statistical analysis and revised the manuscript. All authors read and approved the final manuscript.
